# Estimating Influenza Deaths in Canada, 1992–2009

**DOI:** 10.1371/journal.pone.0080481

**Published:** 2013-11-27

**Authors:** Dena L Schanzer, Claire Sevenhuysen, Brian Winchester, Teresa Mersereau

**Affiliations:** 1 Centre for Communicable Diseases and Infection Control, Infectious Disease Prevention and Control Branch, Public Health Agency of Canada, Ottawa, Ontario, Canada; 2 Centre for Immunization and Respiratory Infectious Diseases, Infectious Disease Prevention and Control Branch, Public Health Agency of Canada, Ottawa, Ontario, Canada; The University of Tokyo, Japan

## Abstract

**Background:**

Poisson regression modelling has been widely used to estimate the disease burden attributable to influenza, though not without concerns that some of the excess burden could be due to other causes. This study aims to provide annual estimates of the mortality and hospitalization burden attributable to both seasonal influenza and the 2009 A/H1N1 pandemic influenza for Canada, and to discuss issues related to the reliability of these estimates.

**Methods:**

Weekly time-series for all-cause mortality and regression models were used to estimate the number of deaths in Canada attributable to influenza from September 1992 to December 2009. To assess their robustness, the annual estimates derived from different parameterizations of the regression model for all-cause mortality were compared. In addition, the association between the annual estimates for mortality and hospitalization by age group, underlying cause of death or primary reason for admission and discharge status is discussed.

**Results:**

The crude influenza-attributed mortality rate based on all-cause mortality and averaged over 17 influenza seasons prior to the 2009 A/H1N1 pandemic was 11.3 (95%CI, 10.5 - 12.1) deaths per 100 000 population per year, or an average of 3,500 (95%CI, 3,200 - 3,700) deaths per year attributable to seasonal influenza. The estimated annual rates ranged from undetectable at the ecological level to more than 6000 deaths per year over the three A/Sydney seasons. In comparison, we attributed an estimated 740 deaths (95%CI, 350–1500) to A(H1N1)pdm09. Annual estimates from different model parameterizations were strongly correlated, as were estimates for mortality and morbidity; the higher A(H1N1)pdm09 burden in younger age groups was the most notable exception.

**Interpretation:**

With the exception of some of the Serfling models, differences in the ecological estimates of the disease burden attributable to influenza were small in comparison to the variation in disease burden from one season to another.

## Introduction

Regression modelling is widely used to estimate the disease burden attributable to seasonal and pandemic influenza [1], with this approach recently endorsed by the World Health Organization (WHO) [2], along with a caution not to compare the number of laboratory-confirmed deaths from the 2009 pandemic with estimates of the number of seasonal influenza deaths. Applying these statistical methods to Canadian data has allowed us to study the full burden of seasonal or pandemic influenza on not only mortality [3], [4], but also admissions to hospital [5], visits to emergency departments [6], and workplace absenteeism [7], thereby gaining a better understanding of the relative risks of severe disease that age and health status poses.

The emergence of a novel influenza A H1N1 strain in the spring of 2009 has resulted in a renewed interest in better understanding the reliability of annual estimates of the number of deaths attributable to influenza [8], [9] amid concerns that methods that assume that all excess deaths are due to influenza are falsely including some deaths which were actually due to other respiratory viruses or environment causes [8], [10], [11]. Much of the concern is directed towards Serfling-style [12] regression models where the seasonality of the weekly baseline is described by a cyclical or sinusoidal function (sine and cosine terms). This baseline is estimated by excluding weeks with influenza activity from the estimation process and then extrapolating the baseline to periods with influenza activity. In addition, it has been noted that some applications of this method have resulted in substantially different estimates of excess all-cause mortality [8]. It is only relatively recently that weekly virological surveillance, such as the Respiratory Viral Detection Surveillance System (RVDSS) of the Public Health Agency of Canada [13] has been available to provide a measure of the weekly level of influenza activity nationally. With the availability of a proxy variable for the weekly level of influenza activity, all weeks can be included in the analysis and the regression model can then simultaneously estimate the seasonal baseline and the number of deaths directly associated with the weekly level of influenza activity. Still, the choice of model parameterization could affect the annual estimates of the number of deaths attributable to influenza.

This study aims to provide annual estimates of the mortality and hospitalization burden attributable to seasonal influenza and the 2009 A/H1N1 pandemic for Canada and discuss issues related to the robustness of these estimates to the choice of statistical model. We applied a variety of Serfling and Poisson regression models incorporating over-dispersion to all-cause mortality and then compared the annual estimates of the number of deaths attributable to influenza. We also estimated and compared the annual estimates for mortality and morbidity stratified by age group, underlying cause of death, hospital discharge status, and primary reason for admission, with the hypothesis that disease severity might be similar enough to induce a strong degree of correlation between these annual measures of disease burden.

## Methods

### Sources of data

**1.1 Mortality data.** Records of deaths occurring in Canada from 1992 to 2009 were obtained from the Canadian Vital Statistics database, Statistics Canada [14], [15] and aggregated to weekly counts stratified by age group and underlying cause of death. Underlying cause of death was coded to the International Classification of Disease [16], [17] version 9 (ICD-9) from 1992 to 1999, and starting in 2000, version 10 (ICD-10). The categories of interest were: respiratory deaths (ICD-9: 460-519 and ICD-10: J00-J99); circulatory deaths (ICD-9: 390-459 and ICD-10: I00-I99) and a third category of ‘other causes’ which included all other causes of death (including external causes). Population denominators for the calculation of rates were obtained from Statistics Canada census and inter-census estimates [18].

For the 2009 pandemic, a total of 428 laboratory-confirmed deaths were reported to the Public Health Agency of Canada [19], though not all of these deaths were certified as due to laboratory-confirmed influenza (J09, J10) as the underlying cause of death [15]. These measures of the pandemic mortality burden were considered in assessing the uncertainty in the estimate of the number of deaths attributable to the pandemic.

**1.2 Morbidity data.** Hospital discharge records from patients admitted to an acute care hospital with a respiratory condition identified in any of the diagnostic fields were extracted from the Canadian Institute of Health Information (CIHI) patient-specific Hospitalization Database (HMDB) [20] for the period September 1994 to March 2010. The HMDB contains all separations (discharge or death) from an acute care hospital anywhere in Canada. Data was abstracted from the patient chart using ICD-9, or ICD-10-CA (Canadian version) [21]. At the 3 character ICD-10 code level used in this study, the ICD-10-CA is consistent with codes used in other countries [22]. The conversion from ICD-9 to ICD-10-CA occurred on a province-by-province basis from 2001 to 2006. Admissions were stratified by age, primary and secondary diagnostic category and discharge status and aggregated to weekly values. An additional subcategory of pneumonia and influenza admissions (ICD-9: 480-487, ICD-10 J09-J18) was also considered. Primary respiratory admissions are defined as admissions with a respiratory condition listed as the primary reason for admission/length of stay. Secondary respiratory admissions are those admissions for other causes with a respiratory condition as a contributing diagnosis.

**1.3 Virological data and proxy variables for influenza activity.** Respiratory virus identifications for influenza A and B and respiratory syncytial virus (RSV) were obtained from the RVDSS, Public Health Agency of Canada [13]. The RSVDSS compiles weekly data from selected laboratories on the number of tests performed and the number positive for influenza, respiratory syncytial virus (RSV), and other respiratory viruses. Specimens are generally submitted to laboratories by clinicians in the course of clinical care, and by clinicians participating in one of our provincial influenza surveillance programs. The predominant testing methods used for influenza detection have varied considerably over the study period and by laboratory. Panel tests were often used.[23]–[25] Though viral identification data has traditionally been used as the proxy variable for influenza activity and has performed well in this capacity [4], once circulation of a novel strain with pandemic potential was announced in late April of 2009, testing increased sharply as did the number of positive tests, and then varied in response to public health needs over the pandemic period. In many jurisdictions, the percent positive remained elevated over an unusually long period of time. As a result, viral identification data was no longer a good measure of the relative level of influenza activity over the pandemic period. Fortunately, with the conversion of hospitalisation coding to ICD-10, patients with laboratory-confirmed influenza were identifiable by the J09 and J10 codes. In addition, the use of J09/J10 (influenza virus identified) admissions to represent the level of influenza activity under ICD-10 coding has improved the model fit in other studies as well as providing better face validity [5], [6]. In order to use a longer study period these two data sources were combined; that is the weekly number of influenza positive tests (prior to the 2007/08 season) and the weekly number of admissions to hospital with laboratory-confirmed influenza for the 2007/08 influenza season through to March 2010 were combined. The splice was positioned to correspond to the ‘flu year’ to ensure separate multipliers for the two data sources.

**1.4 Ethics Statement.** This study was conducted in accordance with the principles expressed in the Declaration of Helsinki Declaration. Data provided by Statistics Canada was collected under the Statistics Canada Act. Data collected by the Canadian Institute of Health Information was provided to the Public Health Agency of Canada and the use of this data was in accordance with data sharing agreements. Data provided by the Public Health Agency of Canada was collected under the Public Health Agency of Canada Act and used in agreement with policy and regulations related to the publication of information related to public health. Identifying formation was not available to this study and most of the data has been previously published. Hence, ethics approval was not required.

**1.5 Data Access.** Access to the data used in this study is available from the data custodians (Statistics Canada, Canadian Institute of Health Information and the Public Health Agency of Canada) as per their data access arrangements. A dataset containing weekly mortality (all cause) and the weekly number of influenza and RSV positive tests has been made available as Table S1 with the description in Table S2.

### Analysis

**2.1 Poisson regression model.** Stratified weekly deaths and admissions were modelled as a function of viral activity, seasonality, and trend using Poisson regression models similar to previously published estimates of the influenza burden in Canada[4], [5], [7]. The regression model was fit using SAS 9.1 [26] PROC GENMOD with a Poisson distribution, linear link function and dispersion parameter specified by:
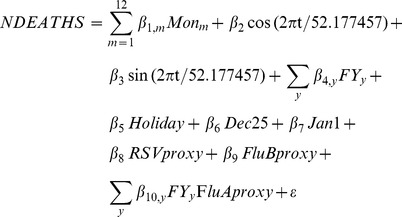
where ***NDEATHS*** represents the weekly number of deaths (or admissions) for the category of interest (for example age group). The monthly indicator variables (*Mon_m_*) and the sinusoidal terms (with *t* = week) are included to account for seasonality. *FY_y_* is a set of indicator variables for each influenza season, *y* (‘flu’ year) starting in September, with an exception for the 2009 pandemic where the ‘flu’ year was defined as May 2009 – March 2010. The β_4_ parameters account for a general trend with more flexibility than the linear, quadratic and cubic trend terms used in the Serfling model below. The variables ***Holiday*** and ***Dec25*** were included in the hospital admission model to account for a reduction in admissions over holidays, and the last week of December (that includes December 25^th^, or Christmas day). The variable ***Jan1*** is an indicator variable for the first week of January, a week when a pronounced spike in respiratory admissions and deaths is often seen. The β_8,_ β_9,_ and β_10, y_ parameters are multipliers for the proxy variables for RSV, influenza B and influenza A respectively. Separate annual multipliers for each flu season are necessary for any meaningful discussion of the annual estimates. Unfortunately, statistical power was insufficient to estimate the annual effects of RSV or influenza B. The ***FluAproxy*** is a combination of laboratory positive tests and admissions to hospital with laboratory-confirmed influenza as described above. The ***FluBproxy*** is the percentage of laboratory tests positive for influenza B. Various alternative parameterizations of the Poisson regression model were included in the sensitivity analysis. In the Week-of-Year Baseline model, all seasonal variables were replaced by a week-of-year indicator variable (53 seasonal parameters). In the Simple Serfling-like Poisson model seasonality and trend terms were replaced by the sinusoidal seasonality (2 seasonal parameters) and linear, quadratic and cubic trend terms as specified for the Serfling model below. The dispersion parameter was included in the model estimation to account for additional variation due to events not captured by the choice of explanatory variables and usually results in larger confidence intervals. A linear link function was used to specify that the weekly number of influenza deaths (or admissions) should be proportional over the year/season to the number of confirmed cases.

Influenza-attributed deaths were calculated as the difference between model-predicted deaths and the model-predicted deaths under the hypothetical absence of influenza, with the latter referred to as the seasonal baseline. Equivalently, the annual estimate can also be calculated by summing the proxy variable over the year/season and multiplying by the corresponding estimated parameter. The former (sum of weekly estimates) is easier to visualise, while the latter approach of working with estimated parameters ensures that the correlation structure induced by the regression model is accounted for. Hence, only disease burden estimates corresponding to an estimated parameter were reported, and confidence intervals for estimates of influenza-attributed rates were calculated from the coefficient of variation of the corresponding multiplier parameter.

**2.2 Serfling regression model.** The percentage of weekly laboratory tests positive for influenza A or B from the RVDSS and the percentage of deaths due to an influenza-like illness (ILI) or influenza (ICD-9: 487, ICD-10:J09, J10 or J11) at various arbitrary threshold levels were used to identify weeks for exclusion from the Serfling models specified by: 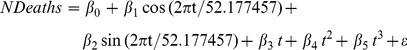
where *t* represents time or week. (Serfling-style models still use regression as the statistical procedure to estimate the parameters. However, these models are characterized by the exclusion of observations during periods of influenza activity.)

The Serfling model was only used in the sensitivity analysis. The annual number of deaths attributable to influenza was calculated for the Serfling models by summing the weekly excess (actual less estimated baseline) over a period that only included the excluded weeks. Both positive and negative ‘excesses’ were included in the sum in order to avoid biasing the estimate upward. For each Serfling model we reported the number of excluded weeks per season, the annual number of excess deaths and the annual baseline for the full year, averaged over 17 seasons (1992/93-2008/April 2009).

**2.3 Cross-Validation and Sensitivity Analysis.** The Poisson model described above was used to calculate annual estimates of the influenza disease burden by categories of interest. These annual estimates were plotted in scatter plots and as a time-series, and the correlation coefficient was used to summarize the disparity between two annualized time-series. Annual estimates for the burden attributed to influenza B were not specifically reported, as only one influenza B multiplier parameter was used for whole study period (and annual estimates calculated from such a model would be perfectly correlated with the annualized proxy variable).

For the sensitivity analysis for all-cause mortality, selected thresholds for the Serfling model and various parameterizations of the Poisson regression model were used to assess the impact of model choice on the point estimate of influenza mortality and its standard error. All parameters included in the main Poisson model described above were statistically significant (with p-values from the type 3 analysis often less than 0.0001).

## Results

**Figure 1:**
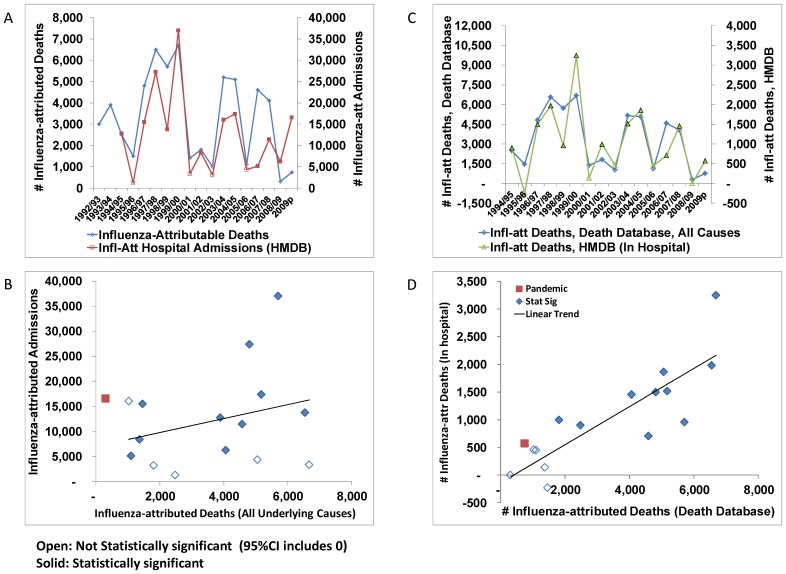
Comparison of annual estimates of deaths and hospital admissions attributable to influenza. Annual estimates of the number of deaths and hospital admissions attributable to influenza are shown as a) an annual time-series with the influenza season identified on the x-axis and b) as a scatter graph of influenza-attributable deaths (x-axis) by hospital admissions (y-axis) with a linear trend identified by the solid black line. Open symbols indicate that the estimate was not statistically significant (95% CI includes 0). In the scatter graph, the A(H1N1)pdm09 estimates are indicated with a red square. c) Annual estimates of influenza-attributed deaths from the death and hospitalization databases are compared in time-series format and d) scatter graph format.

The average number of deaths attributable to seasonal influenza over the 17 seasons prior to the 2009 pandemic was estimated at 3,500 (95% CI 3,200 - 3,700) for an average crude mortality rate of 11.3 (95% CI, 10.5 - 12.1) deaths per 100,000 population per year. These estimates were based on the impact of influenza activity on all-cause mortality. The annual estimates ranged from undetectable to an average of more than 6000 deaths per year over the three A/Sydney seasons (Table 1). Between May and December 2009, an estimated 740 deaths (95%CI, 350-1500) were attributed to the pandemic strain, for a crude mortality rate of 2.2 (95%CI, 1.0 - 4.5) per 100,000 population. The average number of admissions to hospital attributable to seasonal influenza over the 15 seasons prior to the 2009 pandemic was estimated at 12,200 (95%CI, 10,800 - 13,600). The effect of influenza B on hospital admissions was not statistically significant. The annual estimates of respiratory admissions and deaths attributable to seasonal influenza were closely correlated (correlation coefficient of 0.82, see Figure1a, b), as were the mortality estimates from the death and hospitalization databases (correlation coefficient of 0.83, Figures1c, d). The pandemic was a notable exception with a relatively high morbidity burden compared to the mortality burden.

**Table 1:** Estimated Annual Number of Deaths and Hospital Admissions Attributed to Influenza, Canada.

Season	Predominant A Strain and Sub-type	Influenza –Attributed Deaths Canada (95% CI)^2^			Influenza -Attributed Admissions with a Respiratory Dx (95% CI)^2^		
1992/93	A/Beijing/32/92 (H3N2)	3,000	(2,200 – 3,800)				
1993/94	A/Beijing/32/92 (H3N2)	3,900	(3,000 – 4,700)				
1994/95	A/Shangdong/09/93(H3N2)	2,500	(1,700 – 3,300)		12,800	(8,700 – 16,800)	
1995/96	A/TEXAS/36/91(H1N1)	1,500	(200 – 2,700)		1,200	(–4,100 – 6,600)	ns
1996/97	A/Wuhan/359/ 95(H3N2)	4,800	(3,900 – 5,700)		15,500	(11,900 – 19,100)	
1997/98	A/Sydney/05/97(H3N2)	6,500	(5,700 – 7,400)		27,300	(22,900 – 31,700)	
1998/99	A/Sydney/05/97(H3N2)	5,700	(4,800 – 6,700)		13,800	(6,100 – 21,400)	
1999/00	A/Sydney/05/97(H3N2)	6,700	(5,800 – 7,600)		37,000	(32,900 – 41,100)	
2000/01	A/New Caledonia/20/99 (H1N1)	1,400	(500 – 2,200)		3,300	(–750 – 7,400)	ns
2001/02	A/Panama/2007/99 (H3N2)	1,800	(700 – 2,900)		8,300	(2,600 – 14,100)	
2002/03	A/New Caledonia/20/99 (H1N1)	1,000	(–300 – 2,400)	ns	3,100	(–5,700 – 11,900)	ns
2003/04	A/Fujian/411/02 (H3N2)	5,200	(4,000 – 6,300)		16,000	(11,900 – 20,100)	
2004/05	A/Fujian/411/02 (H3N2)	5,100	(4,000 – 6,100)		17,400	(11,900 – 22,800)	
2005/06	A/California/7/2004(H3N2)	1,100	(30 – 2,200)		4,300	(–1,100 – 9,700)	ns
2006/07	A/Wisconsin/67/2005 (H3N2)	4,600	(3,400 – 5,700)		5,100	(380 – 9,700)	
2007/08	A/Solomon Islands/3/2006 (H1N1)	4,100	(2,800 – 5,400)		11,400	(5,200 – 17,700)	
2008/09	A/Brisbane/59/2007 (H1N1)	300	(–800 – 1,400)	ns	6,200	(720 – 11,700)	
2009p	A/California/7/2009 (A(H1N1)pdm09)	740	(350 – 1,500)	ns 1	16,600	(14,100 – 19,000)	
Seasonal Average		3,500	(3,200 – 3,700)		12,200	(10,800 – 13,600)	
	Crude Rate/100,000	11.3	(10.5 – 12.1)		39.5	(34.9 – 44.1)	
	Influenza B (average)^3^	391	(50–770)		1,700	(–270 – 3,580)	ns

Notes:

ns: not statistically significant.

1 Though the model estimate for the 2009 pandemic was not statistically significant based on all-cause mortality, the estimate based on respiratory deaths was statistically significant. Hence, the lower 95%CI was adjusted based on the results of the respiratory model (see Table2).

2 Figures have been rounded.

3 Annual estimates for the burden attributed to influenza B were not specifically reported, as only one influenza B multiplier parameter was estimated for whole study period.

### 1. Pandemic Mortality Burden

Of the 428 deaths with laboratory-confirmed A(H1N1)pdm09 reported to the Agency, 73% were in persons under 65 years of age and 72% were certified as due to influenza as the underlying cause of death. We estimated that 561 (95% CI, 347-775) deaths certified as due to an underlying respiratory cause were attributable to the pandemic. An additional 179 deaths certified as due to other causes were attributed to the pandemic, though this estimate was not statistically significant and the confidence intervals are quite wide. We assumed that the pandemic did not result in fewer deaths from circulatory or other causes, and as a result, set the lower 95% confidence interval (CI) for all-cause deaths attributed to influenza to the lower 95% CI from the respiratory underlying causes model. The resulting estimate is 740 deaths for a rate of 2.2 (95% CI, 1.0, 4.5) deaths per 100,000 population. (For any subsequent analysis, such as for a meta-analysis to assess the pandemic burden on a global scale, the model estimated standard error (coefficient of variation is 0.543) should be used.) The lower 95%CI at 1.0 deaths/100,000 population is close to the figure for deaths certified through ICD-10 coding as due to laboratory-confirmed influenza of 0.9 (Table 2). For persons under 65 years of age, the 216 deaths certified as due to influenza is in good agreement with the 254 (95%CI 192-315) deaths estimate to be attributable to A(H1N1)pdm09.

**Table 2:** A(H1N1)pdm09 Associated Mortality Rates per 100,000 Population, Canada, May – Dec 2009.

Source	Age	# Deaths	Influenza Mortality Rate per 100,000 Population	95% CI (Lower, Upper)		% 65 yrs or older
Laboratory confirmed deaths reported to Public Health Agency of Canada						
	All ages	428	1.27			
	<65	312	1.07			27%
	65+ years	116	2.47			
Underlying cause of death, Vital Statistics Database: Influenza, J09, J10						
	All ages	310	0.92			
	<65	216	0.74			30%
	65+ years	94	2.00			
Influenza-attributed Respiratory Deaths (Regression model estimate)^1^						
	All ages	561	1.7	1.0	2.3	
	<65	254	0.9	0.7	1.1	55%
	65+ years	311	6.6	2.5	10.7	
Influenza-attributed All cause Deaths^2^						
	All ages	740	2.2	1.0	4.5	
	<65	195	0.7	0.7	1.5	74%
	65+ years	540	11.5	2.5	26.1	

Notes:

1 The number of influenza-attributed respiratory deaths was calculated from the regression model for deaths certified as due to an underlying respiratory cause.

2 The estimated number deaths that were attributable to A(H1N1)pdm2009 based on the all-cause model was not statistically significant. However, the estimate based on the respiratory model was statistically significant. Hence, the lower 95% CI for the number of deaths attributed to influenza was set to the lower 95% CI for respiratory deaths attributed to H1N1/p2009, a figure that is close to the number of deaths certified through ICD-10 coding as due to laboratory-confirmed influenza as the underlying cause of death (rates of 1.0 vs 0.92).

Early reports of laboratory-confirmed A(H1N1)pdm09 associated deaths to the Agency suggested that most deaths (73%) had occurred among persons under the age of 65 years of age. However, the results of the regression models suggest that this early estimate was too high as under-ascertainment of the role of influenza was more significant for persons 65 years of age or older (Table 2).

### 2. Annual Estimates of Mortality and Morbidity by Epidemiological Characteristics

**Figure 2:**
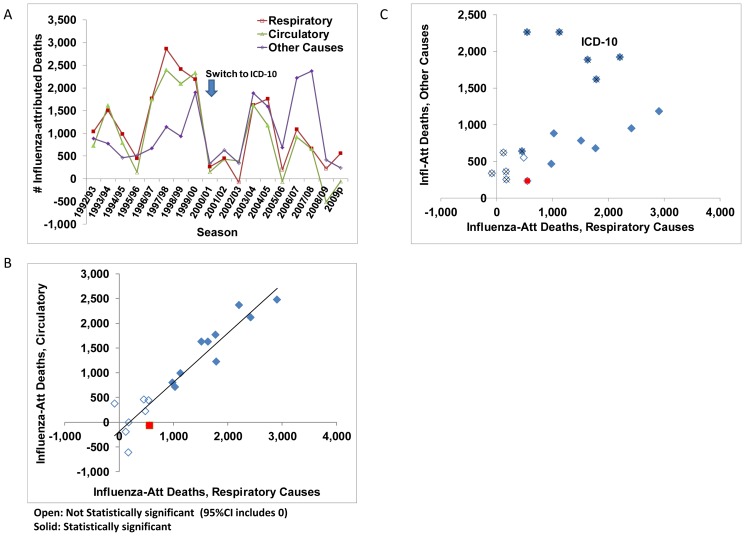
Comparison of the annual estimates of the number of deaths attributable to influenza by underlying cause of death (respiratory, circulatory, and other causes). a) Annual time-series with influenza season identified on the x-axis. b) Scatter graph with a linear trend shown in solid black. Annual estimates based on respiratory and circulatory underlying causes are highly correlated, while in c) a comparison of respiratory to other causes shows a significant change with the conversion from ICD-9 to ICD-10 (denoted by an x). Open symbols indicate that the estimate was not statistically significant (95% CI includes 0). The A(H1N1)pdm09 estimates are indicated with a red square.

**Figure 3:**
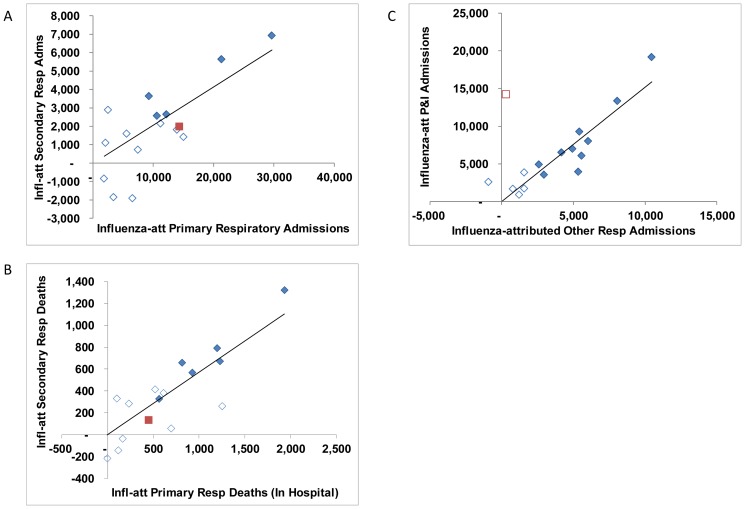
Comparison of annual estimates of the number of hospital admissions attributable to influenza by reason for admission. Open symbols indicate that the estimate was not statistically significant (95% CI includes 0). The A(H1N1)pdm09 estimates are indicated with a red square. A linear trend line is shown in solid black.

**Figure 4:**
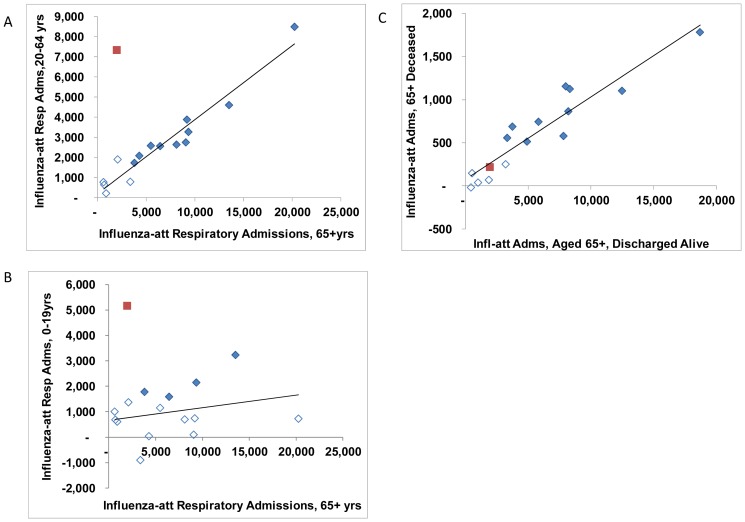
Comparison of annual estimates of the number of admissions attributable to influenza by age groups. Annual estimates of influenza-attributed respiratory admissions for a) the 65+ age group versus 20–64 years age group; b) the 65+ age group versus 0–19 years age group; and c) by discharge status. Open symbols indicate that the estimate was not statistically significant (95% CI includes 0). The A(H1N1)pdm09 estimates are plotted with a red square. A linear trend line is shown in solid black.

Estimates of respiratory and circulatory influenza-attributable deaths were closely correlated (Figure 2a, b), though with the conversion from ICD-9 to ICD-10, the proportion of influenza-attributable deaths coded to other causes increased (Figure 2a, c). Annual estimates of influenza-attributed admissions were correlated by reason for admission (primary versus secondary respiratory *r* = 0.77, Figure 3a), as were in-hospital deaths (Figure 3b). During the pandemic, most influenza-attributable respiratory admissions had a diagnosis of pneumonia or influenza, though for seasonal influenza, annual estimates for pneumonia and influenza versus other respiratory conditions were closely correlated (*r* = 0.91) (Figure 3c). By broad age groups, annual estimates of influenza-attributed hospital admissions were strongly correlated between persons aged 20–64 years and persons 65 years of age or older (Figure 4a). However, the annual disease burden in persons under the age 20 years was a poorer predictor of the burden in adults (Figure 4b). Annual estimates by discharge status (discharged alive versus deceased) for persons aged 65 years or older, were also strongly correlated (*r* = 0.94, Figure 4c).

### Sensitivity Analysis

**Figure 5:**
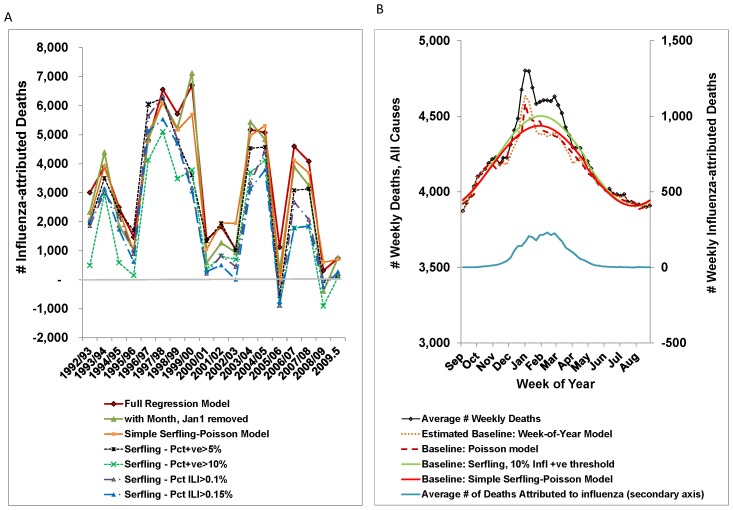
Sensitivity Analysis of selected models to estimate the mortality burden attributable to influenza. a) Poisson regression and Serfling model estimates of influenza-attributable deaths by influenza season. Three parameterizations of the Poisson regression model are shown in solid lines and 4 choices of thresholds for periods of influenza activity for the Serfling model are shown with dashed lines. Serfling models use regression to estimate a cyclical baseline, but exclude weeks with influenza activity. b) Average Number of Deaths (all cause) by Week with the Estimated Baselines for Selected Models. Despite an additional 150–200 deaths occurring in week 1, the averages of the weekly baseline for the full model (dashed line) and the Simple Serfling-like Poisson model (solid red) for January through mid-March were similar. Use of 53 indicator variables – 1 for each week of the year to account for seasonality resulted in a similar weekly baseline (dotted line). The Serfling baseline for a 10% influenza positive threshold is shown in green. The average weekly number of deaths attributed to influenza is shown on the secondary y-axis.

In applying different parameterizations of Poisson regression models to the weekly time-series of all-cause mortality, we found minimal differences in the average number of deaths attributed to influenza and the annual estimates were closely correlated (*r* >0.9 for comparisons of models with annual multipliers) (Table 3 and Figure 5a). Model results using a multiplicative Poisson regression model (log link) rather than an additive model or the negative-binomial distribution in place of the Poisson distribution produced almost identical results (not shown).

**Table 3:** Sensitivity Analysis of the Poisson Regression Models for the Estimation of the Annual Number of Deaths Attributed to Seasonal Influenza.

Model Parameterization	Average Number of Influenza-attributable Deaths	Standard Error	Average Annual Baseline
**Poisson Regression Models with annual multipliers for FluAproxy**			
Full model	3,486	128	216,518
Removed *Jan1*	3,474	131	216,506
Removed *Month*	3,190	131	216,793
Removed *Month, Jan1*	3,122	135	216,850
Removed sine and cosine terms	3,700	130	216,285
Removed *RSVproxy*	3,528	127	216,498
Full model + Annual multipliers for *RSVproxy*	3,306	189	216,590
Simple Serfling-like Poisson model(*FluAproxy, FluBproxy)*	3,517	123	216,530
Simple Serfling-like Poisson model (*FluAproxy)*	3,227	121	216,825
Week-of-year Baseline	3,759	121	216,432
Single multipler for *FluAproxy = %inflApos*	3,082	301	217,043

Though the annual estimates from various Serfling models were closely correlated with the results from the Poisson models (*r*>0.8) they tended to be lower. The source of this difference seems to stem from an over-estimation of the cyclical baseline over the winter months which had been excluded due to influenza activity. We found that excluding fewer weeks (from 33 to 6) increased the estimated baseline (Table 4). Still, simply adding the ***FluAproxy*** variable with individual multipliers for each season (Simple Serfling-like Poisson model) resulted in estimates that were similar to estimates from other Poisson regression models. This effect on the estimated baseline is illustrated in Figure 5b, where all-cause deaths and estimated baselines were averaged by week over the study period.

**Table 4:** Sensitivity Analysis of Serfling Models for the Estimation of the Annual Number of Deaths Attributed to Seasonal Influenza.

	Threshold	Average Number of Influenza-attributable Deaths	Average Number of excluded weeks	Average Annual Baseline
**Exclusion based on the weekly percentage of Deaths due to Influenza or ILI, Death Database**				
	0.03%	2,864	33	217,256
	0.05%	2,767	24	217,300
	0.10%	2,416	14	217,638
	0.15%	2,130	10	217,910
	0.20%	1,911	8	218,135
	0.25%	1,901	7	218,139
	0.30%	1,782	6	218,256
**Exclusion based on the weekly percentage of laboratory tests positive for Influenza A or B**				
	0.5%	2,858	31	217,189
	1%	3,292	27	216,785
	5%	2,894	17	217,145
	10%	1,894	10	218,120
	15%	1,370	6	218,674

## Discussion

Influenza-attributable deaths are deaths that occur in people for whom an influenza infection contributed to the actual death, or at least the time of death, regardless of whether or not influenza was identified as the underlying cause of death. Because of the limitations of an ecological study design, these models have been be given much scrutiny [1], [8], [11], [27], [28]. Some of these reviews have identified poor correlation in published annual estimates of influenza mortality rates, while other studies have found reasonable agreement. Unlike most ecological studies, the peak period of influenza activity in temperate regions occurs over a relatively short period of time, with over 50% of the cases occurring within a 4–5 week period each year[29]. Though influenza was attributed to only 2% of deaths annually, 14–19% of the weekly deaths at the peak for the three A/Sydney seasons (1997/98-1999/00), and up to 10% in other seasons were attributed to influenza. As an ecological study, there is no gold standard for the best model – and the most appropriate model may vary from country to country depending on data availability or unusual events. The short duration and intense effect of influenza on many endpoints provides a unique opportunity to use weekly data to estimate the impact and to use validation and cross-validation techniques at the annual level to assess the internal and external consistency of the annual estimates.

As a measure of external consistency, we assessed the correlation between the annual mortality estimates by cause of death and the annual morbidity estimates by age, reason for admission, and discharge status. Most estimates of the disease burden attributable to influenza were well correlated with only a few exceptions. A notable exception is that the severity of an influenza season among persons under the age of 20 years was found to be a relatively poor predictor of the severity among adults (Figure 4b). The proportion of all deaths that were coded to causes other than cardio-respiratory conditions increased from 54% to 60% with the conversion to ICD-10. However, the conversion had a much larger impact on the proportion of influenza-attributed deaths that were coded to other causes, which increased from 23% to 49% (Figure 2b) for seasonal influenza. Despite the labels of influenza-attributed ‘cardiovascular’ or ‘other causes’, the analysis of in-hospital deaths indicates that the attribution to influenza is limited to patients with a diagnosis of respiratory condition regardless of the certified underlying cause of death. The implication is that the use of all-cause mortality with its wider confidence intervals is a more accurate measure of the full disease burden due to influenza.

For persons over the age of 65 years, the estimated A(H1N1)pdm09-attributed burden was considerably less than for seasonal influenza (Figure 4a, b), however, once admitted to hospital due in part to an influenza infection, the fatality rate for this age group was similar for seasonal and pandemic influenza (Figure 4c). The lower A(H1N1)pdm09 fatality rate for all ages combined is explained by the unusually high number of admissions among persons under the age of 65 years (Figure 4a, b). The low A(H1N1)pdm09-attributed mortality rates in Canada is consistent with the hypothesis of partial prior immunity for persons over the age of 65 which has been confirmed in previous work[5]. Contrary to initial reports where 73% of all laboratory-confirmed A(H1N1)pdm09 deaths reported to the Agency occurred in persons under the age of 65 years, this proportion dropped to 45% for influenza-attributed respiratory deaths and possible much lower for all-cause mortality. The implication is that for hospitalized seniors the pandemic strain was not milder than for seasonal strains and that their infections were less likely to be diagnosed as influenza than for younger persons. Most of the missing pandemic diagnoses were among older patients with pneumonia.

Though no obvious trend was observed over the study period, a full trend analysis was limited by the significant year-to-year variation in the influenza-attributable mortality rate, together with increased virological testing over the study period, and significant coding differences of the underlying cause of death introduced with the conversion to ICD-10.

For internal consistency, we compared the annual estimates of the number of deaths attributable to influenza from different Serfling and Poisson regressions models. The Serfling estimates were dependant on the number of weeks per year that were excluded due to influenza activity, and for most exclusion rules, this method produced lower annual estimates than the Poisson models.

Concern has been expressed that as Serfling models attribute all excess deaths to influenza, that this approach would inherently over-estimated the burden attributable to influenza. It appears that, on the contrary, the fitted cyclical baseline, with only a few data points in the winter months as a guide, tended to overestimate the number of non-influenza deaths in the winter months, and hence underestimate the winter excess due to influenza. Including the proxy variable for influenza activity along with annual multipliers for independent estimates of the annual excess was sufficient to stabilize the annual estimates.

Additional parameters that allowed the baseline to adapt more closely to the weekly data had little effect on the average annual estimates for influenza mortality. For example, deaths in 1^st^ week in January were elevated by an estimated 163 deaths (95% CI 88-240). Removing this parameter from the regression model resulted in these additional deaths being absorbed into the baseline estimates for other weeks. The same was observed for the monthly indicator variables and the RSV proxy variable. Even using a week-of-year indicator variable (53 parameters) produced similar results. On the other hand, removing all parameters related to seasonality resulted in a two-fold increase in the estimated number of influenza-attributed deaths (not shown).

Some earlier models for estimating the burden of influenza mortality used the percentage of tests positive for influenza as the proxy variable for influenza activity and the same multiplier for each season [30]. Including only one multiplier for influenza activity forces the annual mortality estimates to be proportional to the annualized proxy variable and produced annual estimates that were poorly correlated with the estimates for the full model and were also about 10% lower (Table 3).

Our sensitivity analysis was limited in the breadth of comparisons included. Other options for proxy variables are emerging [31] and have not been evaluated in this study. While we included a proxy variable for RSV activity, many other respiratory viruses are now under national surveillance. In addition, our sensitivity analysis was limited to assessing the performance of different models for all-cause mortality only. As the annual estimates of influenza-attributed admissions for children and adults were poorly correlated, further assessment, particularly of models using a single influenza multiplier for more than one season is warranted for other time- series. The assumption that testing was consistent over each season prior to the pandemic seems reasonable based on a previous study of the empirical epidemic curves derived from this data [29].

Some of the annual influenza disease burden estimates (or the lower 95% confidence interval) in the tables or figures are negative. Negative estimates will occur when the true number of events (deaths or admissions) due to influenza is less than the natural variation in the total number of events (all-cause mortality or hospital admission) during periods of influenza activity. For the Serfling estimates, we summed all positive and negative weekly ‘excesses’ over the defined influenza period in order not to introduce biases by ignoring negative estimates. Technically, though plausible in a few rare instances, it is unlikely that an influenza infection ‘saved’ lives, and hence any negative ‘excess’, is most likely due to the random variation of the total number of deaths. Hence, to avoid biasing the annual total, these natural or random dips below baseline must be included to balance out the nature swings above baseline.

Though Poisson regression, or equivalently, negative binomial regression models with annual multipliers to adjust for potential year-to-year differences in the relationship between the proxy variable for the level of influenza activity and the resulting disease burden are emerging as the preferred model, various methods have been used internationally to estimate the A(H1N1)pdm09 mortality burden. A recent review article [32] summarizes many of the international studies to date. As noted in our study, a higher disease burden for pandemic versus seasonal influenza among persons under 65 year of age was a common experience in most countries [32]. Published international estimates of the A(H1N1)pdm09-attributed mortality rate for 2009 have varied. Some of the variation is likely real with higher rates of 11 and 10 per 100 000 population for Mexico (using all-cause mortality) [33] and China (using respiratory and cardiovascular deaths) [32] respectively. Choice of methodology can influence the estimates with one study estimating an influenza-attributable mortality rate of 0.8 per 100,000 for the United States [34] and another by Charu and colleagues at 4.7 (95%CI 2.0 – 7.5) [35]. The lower estimate was produced using a Serfling model with monthly data for pneumonia and influenza deaths, while the latter study used a methodology similar to ours. The estimated mortality rates from the Charu study are similar to ours at 2.6 (95%CI 1.5 – 3.8) compared to 1.7 (95%CI 1.0 – 2.3) for respiratory causes, and in both cases the estimate for circulatory causes was not statistically significant. Though the Charu estimate for cardio-respiratory causes was twice our all-cause estimate, this difference was not statistically significant.

For future estimates of influenza disease burden, increases in virological testing of hospitalized patients along with the ICD-10 coding to identify laboratory-confirmed cases will offer significant improvements in the measurement of influenza activity specific to the region and age group. With a better influenza proxy variable, the expectation is for a slight increase in the estimates of disease burden attributed to influenza. It is therefore important that estimates based on new models forms or even a new parameterization of the regression model be compared head-to-head with a Poisson regression model that includes at least seasonality, secular trend and a proxy variable for influenza or influenza A with annual multipliers and a dispersion parameter or equivalently the use of the negative binomial distribution.

In summary, differences in the ecological estimates of the disease burden attributable to influenza were small in comparison to the variation in disease burden from one season to another. Poisson regression models that included at least seasonality, a secular trend and a proxy variable for influenza or influenza A with annual multipliers and a dispersion parameter to correct for overdispersion produced reasonably consistent estimates of the annual mortality burden attributable to influenza and these estimates were highly correlated with annual estimates for the morbidity burden for adults. The annual morbidity burden for children (under the age of 20 years) was found to be a poor predictor of the corresponding adult burden. Influenza continues to be a significant contributor to mortality.

## Supporting Information

Table S1 **Database containing weekly time-series of all-cause mortality and viral identifications.**
(XLSX)

Table S2 **Description of variable names.**
(XLSX)
